# Evaluating Flexural Performance of Repaired Reinforced Concrete Beams under Static and Repeated Loading Using Non-Destructive Methods

**DOI:** 10.3390/ma15186276

**Published:** 2022-09-09

**Authors:** Boyu Wang, Rishi Gupta

**Affiliations:** Department of Civil Engineering, University of Victoria, 3800 Finnerty Road, Victoria, BC V8W 2Y2, Canada

**Keywords:** flexural repair, non-destructive testing, repair materials, repeated loading

## Abstract

Deterioration of concrete structures is one of the major issues faced by the construction industry. Repair and rehabilitation are necessary to extend the service life of such structures. This study aims to investigate the effect of repair material type, length of repaired region, and loading regime on the structural characteristics of the repaired reinforced concrete (RC) beams. To achieve this goal, a total of 30 repaired and non-repaired RC beams were prepared and tested under static and repeated loading conditions. Three types of sounding-based non-destructive test (NDT) methods are employed to determine the material deterioration and sub-surface delamination after repeated loading. Results showed that under static loading conditions, full-length repaired beams had better performance than 1/3-span repaired beams. Beams repaired with cementitious repair mortar containing modified binder and cementitious repair concrete in full length had a yield strength that was 14% and 9%, respectively, higher than that of beams repaired in 1/3 span. All RC beams with full-span repair outperformed the intact beams. After repeated loading, beams repaired with cementitious repair with modified binder over full length showed a 14% improvement in yield strength compared to control samples. It was found that repair materials that had a high compressive and flexural strength are beneficial. The resonant frequency drops correlate well with the yield strength results. The formulas proposed by Canadian Standards Association (CSA) 23.3 can effectively predict the moment resistance of both intact (control) and repaired RC beams. The ratio of experimental moment resistance values to its predictions ranges from 0.91 to 1.04.

## 1. Introduction

Existing reinforced concrete (RC) structures are subjected to structural deterioration due to their intrinsic defects (such as poor-quality concrete), external loading, and adverse environment [1]. In recent years, many materials or methods have been proposed to repair and strengthen RC structures to extend and retain their service life. They include the use of strain-hardening cement-based composite [2,3,4], polymer-modified cementitious mortar [5], geopolymer mortar [6], ultra-high performance concrete [7,8], and fiber-reinforced polymer sheets [9,10,11]. Various repair products can be classified into three main categories including cement-based materials, polymer-modified cement-based materials, and polymer or resin materials [12]. The selection of repair materials is affected by the factors such as compatibility between repair and parent materials, durability, and bond strength of the repair materials [13]. Different repair materials have their own advantages and limitations. Portland cement mortar or concrete is one of the most common repair materials used because of its availability and low cost. However, under adverse environmental conditions, the performance of Portland cement-based products has been less than satisfactory, especially in aggressive exposure environments [14]. As a result, polymer modifiers such as acrylic, styrene butadiene, and some co-polymers have been added to modify the cement latex. The cement-based repairs with modified binders are found to have improved bond strength, compressive, and tensile strength [15], reduced shrinkage [16], and permeability [15]. However, there is limited work that reported the performance of steel reinforced concrete structures repaired by cementitious materials with modified binders.

For actual structures, such as bridges, repeated loading simulates the real loading situation more closely than static loads. As a result, it is important to ensure that repaired structures have sufficient load-bearing capacity under both static and repeated loading conditions. Many studies focused on studying the static and fatigue loading performance of damaged RC beams strengthened with fiber-reinforced polymer [17,18,19,20] and ultra-high-performance concrete sheets [13,21]. The strengthening sheets made of polymers and cementitious composites are externally bonded to the tension side of RC beams with and without adhesives. The load-bearing capacity of the damaged structures was improved due to the increased cross-sectional area introduced by the externally bonded sheets. The effectiveness of using strengthening sheets is dependent on the use of sheet anchors [17], number of sheet layers [19], size of repair [21], and material properties of sheet layers [13]. Most studies [17,18,19,20,21] have found that externally bonded layers can effectively recover the load-bearing capacity of damaged beams and in some cases [20], the strengthened beams outperformed the undamaged beams. However, using externally bonded sheets could be limited when the deteriorated concrete and corroded steel reinforcement need to be removed prior to repair. The Guide To Concrete Repair [22] stipulates a series of steps for repair which involves saw cutting the perimeter of the repair area, removing the deteriorated and damaged concrete/steel, and then replacing it with different types of repair products. This repair technique does not increase the cross-sectional area of the repaired structures, which makes it different than using externally bonded sheets. Safdar et al. [23] investigated the flexural behavior of RC beams with part of the beams retrofitted with ultra-high performance fiber reinforced concrete (UHPFRC). It was found that the use of UHPFRC led to higher structural stiffness and delayed the formation of macro cracks. Teixeira et al. [24] conducted an experimental study that used a polymer-modified mortar and alkali-activated mortar to repair damaged RC beams. The repaired RC beams were prepared with a repaired region that accounts for 1/3 and 3/3 of the span. It was found that the repair length and type of repair materials affected the yield and ultimate strength. However, authors could not find research that studies the flexural behavior of such repaired RC beams under repeated loading. In a real loading condition, repaired beams are expected to experience repeated load at different amplitude. It is of interest to evaluate the progressive damage and bond performance of repair after repeated loading.

During repeated loading, it is important to monitor and assess the progressive damage that occurred within repaired RC structures. Most non-destructive test (NDT) methods can be classified as either a local or global damage identification technique [25]. The local damage identification techniques such as ultrasonic pulse velocity (UPV) method may require prior knowledge of approximate defect locations as testing the entire structure could be time-consuming. The UPV is dependent on the density and elasticity of the materials. Because of that, the UPV method is useful in detecting uniformity and determining the strength and mechanical properties of concrete [26]. The global damage identification technique such as vibration-based methods [27] examines changes in global vibration characteristics of structures and gives holistic evaluations on structural health. Modal parameters such as natural frequencies, mode shapes, and their variants are commonly used as damage indices. Capozucca [28,29] found that the drop in natural frequencies of RC beams can well reflect the damages such as cracks and stiffness loss as a result of progressive damage. During repeated loading, it has been found that beams with cold-join interfaces may fail along with the cracking of concrete itself. Shah et al. [30] reported that the number of load cycles are high for intact specimens but decrease with the mismatch in elastic modulus of either side of the interfacial materials. Similar work was reported by Wang and Gupta [31,32] that the concrete-repair interface at the perimeter of the repair area was prone to fail under flexural static and cyclic loading. The modulus of rupture of the repaired beam had approximately 50% of that of the intact beams. It can be critical to not only detect cracking but also the interfacial cracking between concrete repair and the substrate during repeated loading.

In summary, most previous studies focused on the static and cyclic loading performance of strengthened RC beams with externally bonded layers. However, scare studies reported cyclic loading performance of beams where deteriorated materials are replaced by repair patches. It is of interest to study the bond between the repair and parent concrete and investigate the progressive damage after repeated loading and unloading. This study aims to study the mechanical properties of the repaired RC beams under static and repeated loading performance as compared to the control beams. The RC beams are repaired using two types of commercially available repair products over the middle third and full length. Three sounding-based NDT methods are used to detect the progressive damage with repeated loads. The effect of repair length, bond strength, and compressive strength of the repair on the mechanical performance of repaired RC beams will be investigated. The models proposed by the current codes and standards are compared with the experimental results in this study. This research found that formulas proposed by code Canadian Standards Association (CSA) 23.3 can effectively predict the moment resistance of both intact and repaired RC beams based on the results of 12 repaired RC beams. The ratio of experimental moment resistance values to its prediction ranges from 0.91 to 1.04. The repair material that has higher bond strength and flexural strength is found to be beneficial to a more successful repair. The findings of this study will contribute to better utilization of concrete repair for designing and developing more durable repaired RC structures.

## 2. Materials and Methods

### 2.1. Materials

The materials used in this study involve one lab-developed normal strength concrete mix and two types of commercially available repair materials that are widely used in the field of concrete structures restoration. The normal strength concrete (termed as Mix S) serves as the parent material to receive the repair. Mix S is made of Type I cement, sand, gravel, superplasticizer, air entraining admixture, and water with a target strength of 45 MPa. According to the product specification, the HWRA complies with ASTM C494/C494 M [33] requirements for Type A, water-reducing, and Type F, high-range water-reducing admixtures. The AEA complies with the requirements of ASTM C260/260 M [34]. The sand and gravel are supplied by local BC vendors, and they have fineness moduli of 2.85 and 6.60, respectively. The detailed mix proportions of Mix S are listed in Table 1. The two types of repair materials used in this study include cementitious repair concrete (Mix F) and cementitious repair mortar with modified binder (Mix M). Per bag of pre-mixed Mix F and Mix M, 2.5 kg and 2.37 kg of water are added following the recommendations from the manufactures. The water-to-material ratio (*w/m*) for Mix S, F, M are 0.08, 0.1, and 0.09 respectively. Table 2 summarizes the fresh and hardened properties of the three repair mixes. The setting time information for each mix is obtained from the product brochure provided by the manufacturers. The air content and slump values are determined as per ASTM C231 [35] and ASTM C 143 [36], respectively. The compressive strength ($f_{c}^{\prime }$) is measured on samples after 28 days of curing in water at 21±2 °C in accordance with ASTM C39 [37]. The results for slant shear bond and splitting tensile bond tests are adopted from a previous study [31] conducted by the authors. More information about the properties such as freeze-thaw, cyclic loading, and corrosion resistance of the repair materials can be found in Wang et al. [31,32,38] and Bajaj et al. [39]. However, due to the proprietary nature of the repairs, the detailed information about materials’ constituents is not available.

### 2.2. Test Specimens

A total of 30 RC beams are constructed and used for evaluating the flexural behavior of repaired RC beams under static and repeated loading. Six of them are control beams without repair while 24 beams are repaired beams using the two types of cement-based repair materials. Table 3 summarizes the specimen, and the loading protocol details.

**Figure 1:**
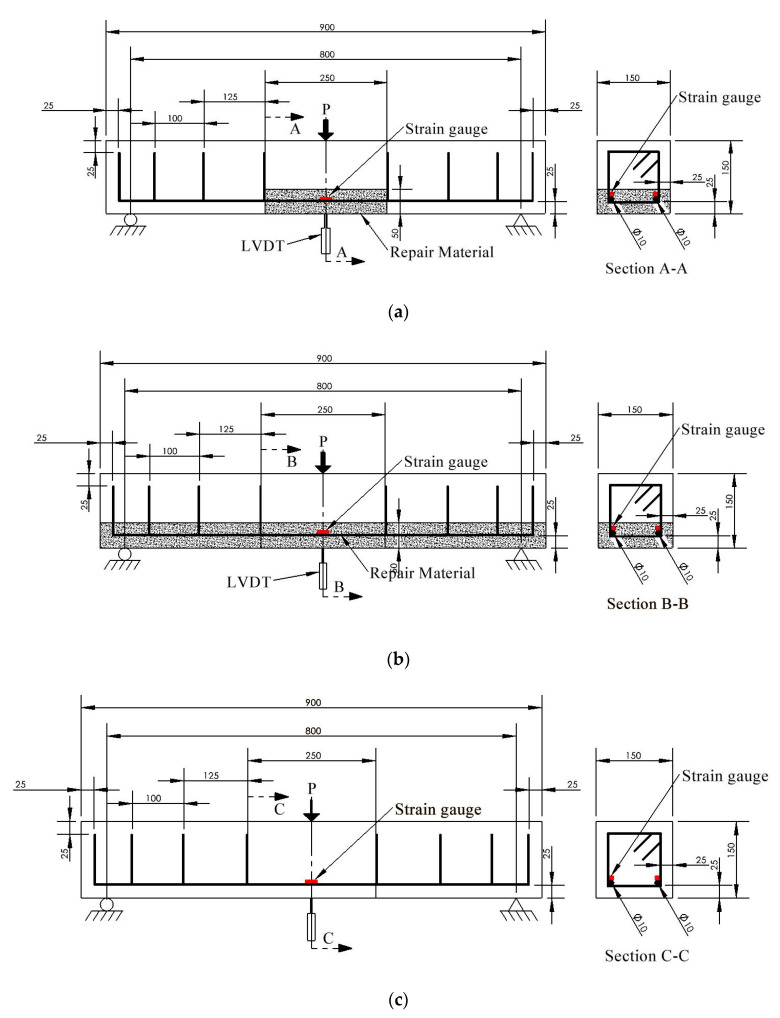
Steel reinforcement details for (**a**) RC beam with 1/3-span repair (**b**) RC beam with full-length repair (**c**) Intact beam without repair (all dimensions in mm).

Six control beams labeled as “Ctrl_st” and “Ctrl_r” are tested under static and repeated loading, respectively. Twenty-four repaired beams are divided into 8 groups in accordance with repair length, repair material type, and loading protocol to cover various combinations for testing. Each group consists of three identical RC beams to have good confidence in test data. The naming of sample ID follows the rule: repair material_repair_length_loading protocol. For example, sample ID “F_1/3_st” represents that RC beams are repaired with Mix F, have a repair length of 1/3 span, and are tested under static loading. The dimension of the RC beams is shown in Figure 1. Figure 1a–c shows the beam with 1/3-span repair, the beam with full-length repair, and the intact beam (control beam), respectively. The length of all RC beams is 900 mm with a rectangular cross section of 150 mm by 150 mm. Two rebars with 90° hooked ends are placed on the tensile side of the beam serving as longitudinal reinforcements, as shown in Figure 1. Six stirrups were tied to longitudinal bars to prevent shear failure, and they have a maximum spacing of 125 mm. The 10 M/400 W black steel bars are used for making stirrups and longitudinal bars, which have a nominal diameter of 11 mm and yield strength of 400 MPa. The repair section has a thickness of 50 mm (2 inches) and a width of 250 mm (1/3 span) or 900 mm (full length) as shown in Figure 1a,b.

### 2.3. Specimen Preparation Procedure

**Figure 2:**
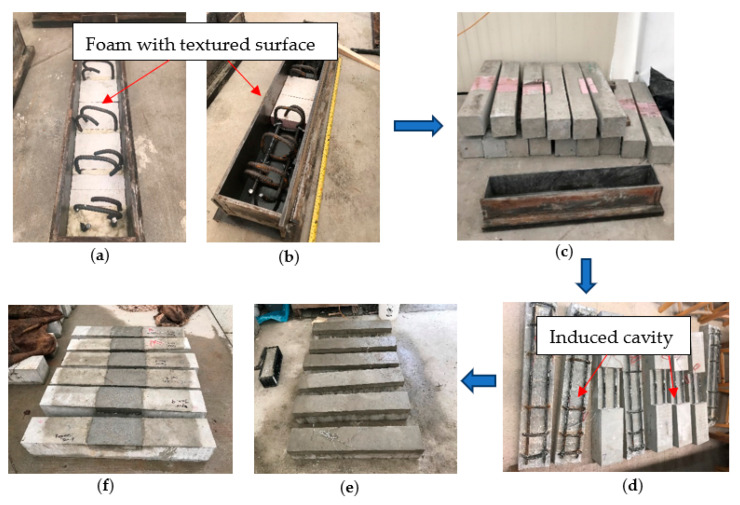
Sample preparation procedures (**a**) Inserting foam for full-length repaired beams (**b**) Inserting foam for 1/3-span repaired beams (**c**) demolded samples (**d**) Samples with foams removed (**e**) Full-length repaired beams (**f**) 1/3-span repaired beams.

There are numerous examples in the field such as bridge decks or parking garages, where deteriorated concrete on the tension side is removed, parent substrate cleaned, and prepared to receive the repair materials. To simulate the in situ repair conditions, prismatic beams with an induced cavity are cast. The fabrication procedure is shown in Figure 2. Foam pieces are placed in the mold before concrete placement and then removed to induce a cavity. Textured papers are attached to the top surface of the foams. The textured papers have similar surface roughness as that of surface profile chip #6, which according to ICRI Technical Guideline No. 310R-2013 [40], simulates the surface profile on site for parking garages, as shown in Figure 2a,b. The benefit of using foam blocks with textured paper is that it can remove any variations associated with preparing the surface that receives the repair. After concrete placement in molds, according to ASTM C31/31M [41], five seconds of vibration using an internal vibrator is performed to consolidate the concrete. The specimens are kept at the ambient environment (15±5 °C with 80–90% relative humidity) for 48 h before demolding. After demolding (as shown in Figure 2c), they are transferred to a water bath. Water curing temperature is maintained at 23±2 °C, and all specimens are cured for 7 days followed by a 28-day air curing at ambient environment (15±5 °C with 80–90% relative humidity). The inserted foams are then removed (as shown in Figure 2d), and the specimens are repaired with Mix F and Mix M, as shown in Figure 2e,f. The repaired RC beams are left at ambient environment (15±5 °C with 80–90% relative humidity) for 2 days and cured for 28 days before testing. Specimens are also constructed as control with no cavities and have the same curing time and condition as that of repaired samples.

### 2.4. Test Setup and Instrumentation

#### 2.4.1. Strain Measurement

Due to the large number of samples used in this study, only 1 out of 3 beams with the same sample ID (as shown in Table 3) has strain gauges attached. Two strain gauges are attached to each repaired beam. They are bonded to the bottom surface of the longitudinal bars in the middle of the beam. 

#### 2.4.2. Loading Protocol

A three-point bending test is performed on RC beams using a Materials Testing System (MTS) with a load bearing capacity of 200 kN. Specimens were tested under both static and repeated loading. Under static loading, all specimens were loaded at a rate of 1 mm/min until the yield stress of the steel is met and then at a rate of 2 mm/min until the crushing of concrete is observed in the compression zone.

**Figure 3:**
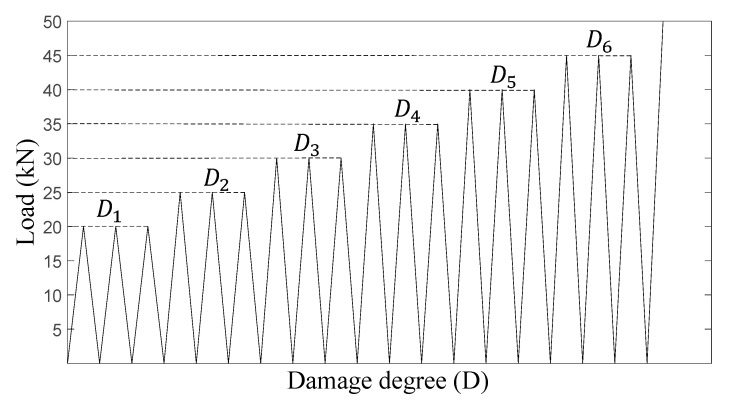
Loading-unloading regime of the repeated loading.

During repeated loading, the test protocol consists of a sequence of increasing load at an equal interval of 5 kN. Figure 3 illustrates the loading and unloading regimes that are adopted in this study. The maximum loads per cycle applied are 20 kN, 25 kN, 30 kN, 35 kN, 40 kN, 45 kN, which corresponds to different damage degrees, $D_{i}$ (*i* = 1–6), of the beams after the loading. The beams are first loaded at a rate of 1 mm/min until the maximum load is reached and unloaded at the same rate until the load is almost zero. The loading–unloading process is repeated three times per load value before moving to the next damage degree. Between each damage level, NDTs are performed which include hammer percussion, modified chain drag, and ultrasonic pulse velocity tests, which are described in the following sections. One linear variable displacement transducer (LVDT) is mounted at the middle span of the beam to record displacement, as shown in Figure 1. The displacement values are used to characterize the elastoplastic properties of the RC beams. After repeated loading (damage degree $D_{6}$), beams are loaded until failure at a rate of 1 mm/min.

#### 2.4.3. Non-Destructive (NDT) Methods

Three types of sounding-based methods are employed to determine the material deterioration and sub-surface delamination after repeated loading. One of the standard methods to determine delamination is outlined by ASTM D4580 [42] and is called chain drag test, which has been widely used in the field. According to ASTM D4580, a heavy-duty chain or a hammer can be dragged over a concrete surface and the detection of delamination occurs by the test performer hearing dull or hollow sounds. However, this test is adversely impacted by the subjectivity of the test performer. To overcome this issue, in the current study, the sound of the impact is recorded by a microphone, and the frequency spectrum of the impact is extracted and used for defect identifications. Both hammer and chain are used to impact the concrete. Another sounding method called ultrasonic pulse velocity (UPV) is used to compare with the results measured by the previous two methods. The steps to perform the UPV test is outlined in ASTM C597 [43]. The detail for performing each test is explained in the following section.

##### Hammer Percussion Method

**Figure 4:**
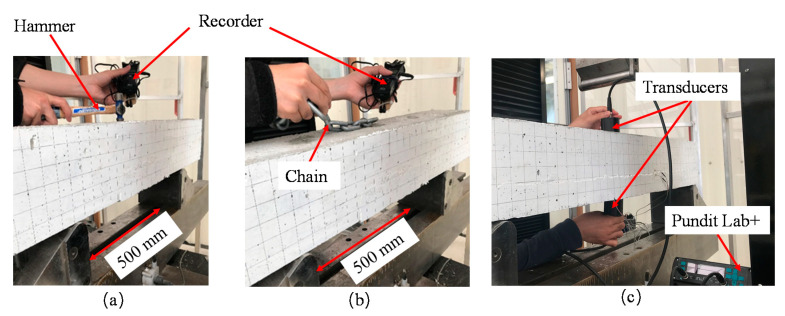
Experimental setup of (**a**) Hammer percussion test (**b**) Modified chain drag test (**c**) Ultrasonic pulse velocity test.

The hammer percussion test [44] is carried out using a 57 g (2 oz.) spherical head steel hammer striking the center of the beam to introduce low-amplitude vibrations of the beam, as shown in Figure 4a. The generated beam vibrations were detected by means of an acoustic recorder and microphone. The recorder has a sampling rate of 96 kHz at 24-bit depth, which indicates a working frequency range from 0 to 48 kHz. The microphone recorded vibration signals from two microphones, and the signal collected from the microphone that is closer to the concrete surface is used for frequency analysis. The recorder exported audio files in WAV format. Audacity audio software was used to trim the vibration signal such that the environmental noises before and after the impact can be removed. MATLAB software was used to perform a single-sided fast Fourier transform on the WAV files to extract frequency information.

##### Modified Chain Drag Test

The modified chain drag test involves dragging a heavy-duty chain with four links over the concrete surface and collecting vibration responses of the beam using a digital recorder, as shown in Figure 4b. The chain link has a diameter of 9.5 mm (3/8 inch). The vibrational frequency of the beam is extracted from the recorded audio following the same data processing procedure of the hammer percussion test. Compared with the traditional chain drag test standardized by ASTM D 4580 [42], the modified chain drag removes the subjectivity of the operator variance from the test. 

##### Ultrasonic Pulse Velocity (UPV)

UPV tests are executed using Proceq Pundit Lab^+^ device. Transducers are placed at the center of the beam such that the ultrasonic pulse can travel across the repair and its interface, as shown in Figure 4c. The direct arrangement of the sensors is used to ensure the maximum signal transmission between the transducers. The transducer frequency was set as 125 kHz such that the wavelength of the ultrasonic wave is at least twice as large as the aggregate size and rebar diameter, which is recommended by the equipment supplier.

## 3. Results

### 3.1. Failure Mode and Crack Pattern

**Figure 5:**
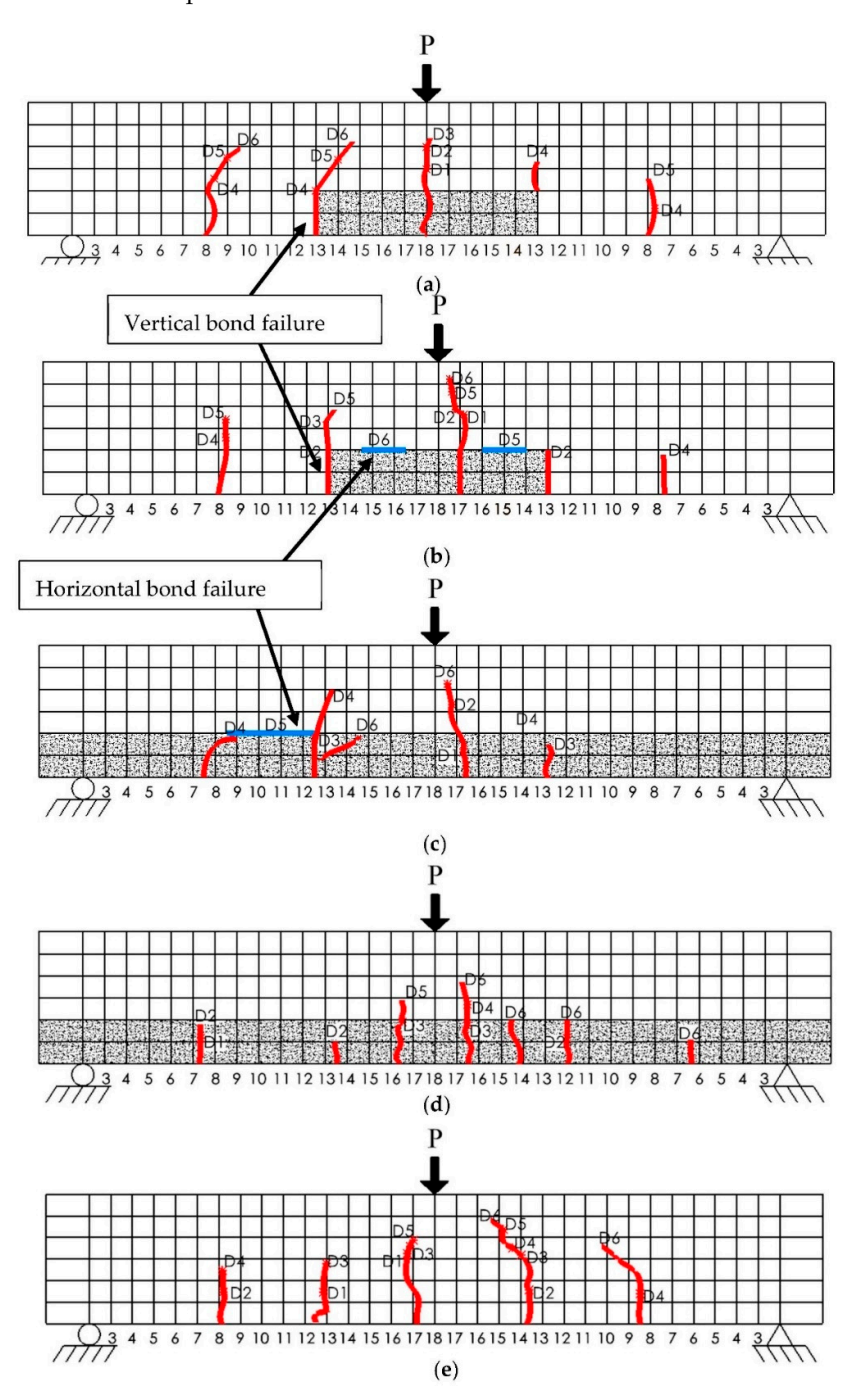
Crack pattern of beams with (**a**) 1/3-span repaired with Mix M (**b**) 1/3-span repaired with Mix F (**c**) full-length repair with Mix F (**d**) full-length repair with Mix M (**e**) no repair.

**Figure A1:**
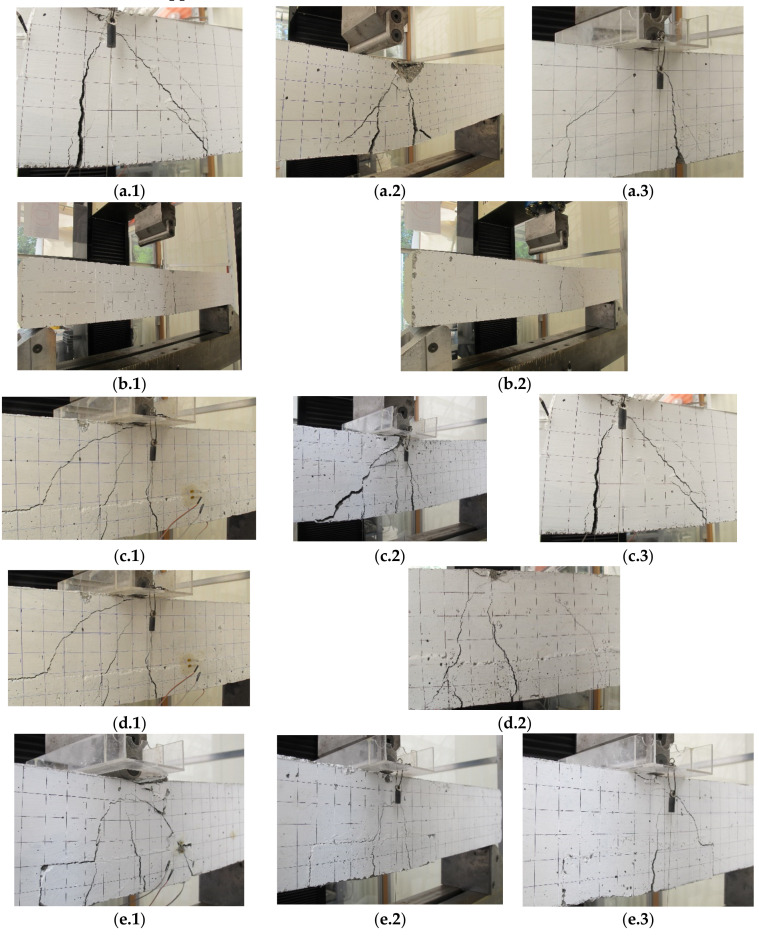

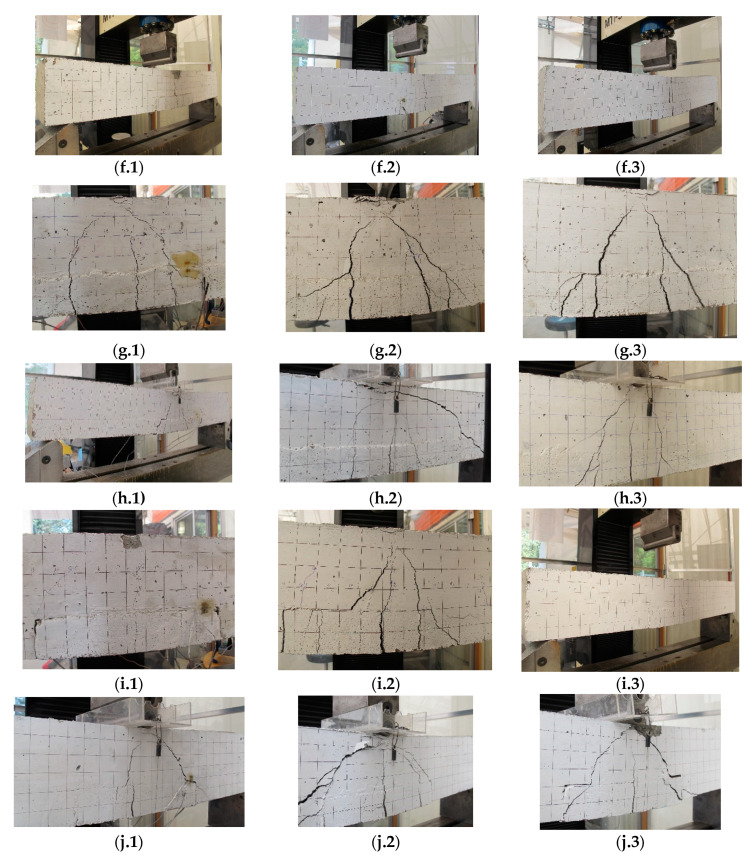
Failure Crack Patterns: (**a.1**–**a.3**) Control samples 1–3 under static load (**b.1**,**b.2**) Control samples 1–2 under repeated load (**c.1**–**c.3**) Mix F sample 1–3 repaired in full length under static load (**d.1**,**d.2**) Mix F samples 1–2 repaired in full length under repeated load (**e.1**–**e.3**) Mix F samples 1–3 repaired in 1/3 span under static load (**f.1**–**f.3**) Mix F samples 1–3 repaired in 1/3 span under repeated load (**g.1**–**g.3**) Mix M samples 1–3 repaired in full length under repeated load (**h.1**–**h.3**) Mix M samples 1–3 repaired in full length under static load (**i.1**–**i.3**) Mix M samples 1–3 repaired in 1/3 span under repeated load (**j.1**–**j.3**) Mix M samples 1–3 repaired in 1/3 span under static load.

All beams were uncracked before testing. During repeated loading and unloading, noticeable crack initiation and propagation were observed on the beams. Figure 5 shows the typical failure crack pattern of the repaired and control beams. The red color indicates the vertical cracks while the blue color represents the horizontal interfacial cracks. The symbol next to the cracks shows the damage level at which the cracks started. As shown in Figure 5a,b, the vertical bond failure initiated when the damage degree reaches $D_{4}$ and $D_{2}$, respectively, for samples with a 1/3 span repaired by Mix M and Mix F. Table 4 summarizes the information about when the bond failure started in the vertical and horizontal direction. As shown in Table 2, Mix M showed higher slant shear and splitting tensile strength, which potentially delay the bond failure between Mix M and the parent material. As shown in Table 4, it is observed that all 6 samples with a 1/3-span repair had vertical interfacial cracks. Similar findings were reported by Wang and Gupta [31] that all repaired beams (without reinforcement) failed at the vertical concrete-repair interface. Figure A1 in Appendix A shows the crack patterns after failure of all beams.

To quantify the progressive damage, the crack length of beams at each damage level was measured and summarized in Table 5. Note that the crack length includes both cohesive cracks and interfacial cracks that occurred at the concrete-repair interface. As shown in Table 5, at damage degree $D_{6}$, beams with a full-length repair by cementitious repair mortar with modified binder (Mix M) showed the lowest average crack length among all mixes. In comparison, the control mix showed the longest average crack length which is 556 mm.

### 3.2. Load-Deflection Behavior

#### 3.2.1. RC Beams under Static Load

**Figure 6:**
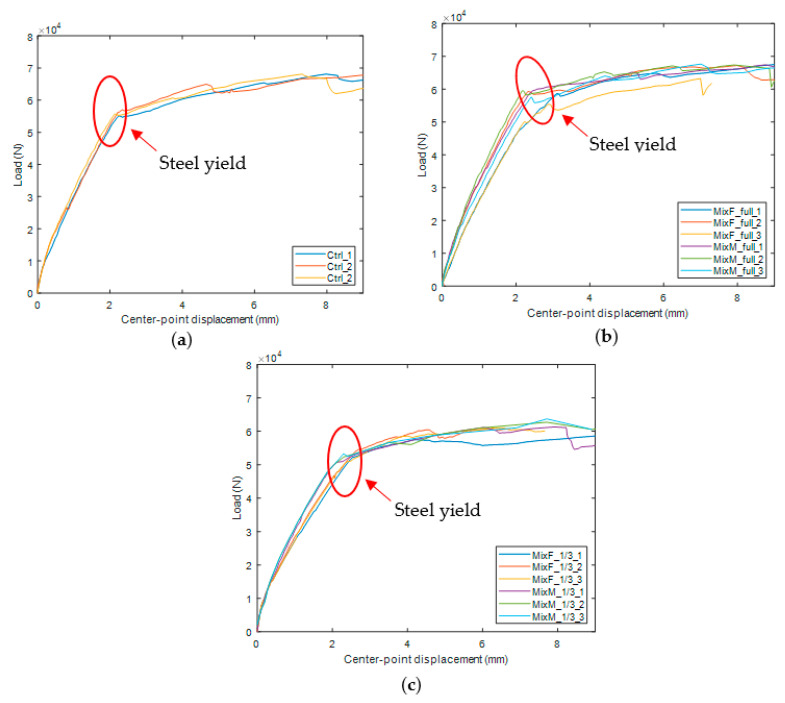
Load-center point deflection curve of beams with (**a**) 1/3 span repair (**b**) full-length repair (**c**) no repair.

Figure 6a–c shows the load vs. center-point deflection curves for repaired and intact (control) beams.

Table 6 summarizes the yielding loads and NDT test results. The yield strength is measured based on force-displacement curve. Compared with the intact beams (control), the average yield strength of beam M_full_st and beam F_full_st was 6.4% and 3.6% higher, respectively. Compared with beams having 1/3-span repairs, the yield strength of beams having a full-span repair using Mix M and Mix F is 14% and 9%, respectively, higher. Khan and Abbass [2] found that the use of a layer of steel fiber-reinforced concrete on the tension side is beneficial to improving yield strength of the steel rebar. In the case of this study, Mix M has the highest flexural strength (7.0 MPa, as shown in Table 2) which helps increase the yield strength of Mix-M repaired beams. The yield strength of beam F_1/3_st and M_1/3_st was 5.3% and 6.9% lower than that of the control beam. During the static loading, interfacial cracking was observed. According to a previous study by Wang and Gupta [31], interfacial cracks between concrete and repairs could lead to as high as 50% loss in flexural strength for unreinforced concrete. We can see that such effect is mitigated in the case of RC beams as steel is carrying most of the tension forces. In terms of resonant frequency (RF) results, beams with higher yield strength tend to have a higher resonant frequency, whereas UPV results for all samples have no significant difference.

#### 3.2.2. Beams under Repeated Loading

Table 7 summarizes the yield strength of beams after going through 6 groups of loading–unloading cycles. The control mix samples experienced a loss of 4.9% in yield strength after repeated loading whereas the yield strength of other samples is comparable to that before repeated loading. This manifests that both Mix M and Mix F have better cracking resistance performance under repeated loading compared to the control mix. Because the repeated loading stress is smaller than the yield stress of steel and the number of cycles is much less than the fatigue cycles of the steel, so the steel did not experience much damage as opposed to concrete. It should be noted that beams with full-length repairs have higher yield strength compared to beams with a 1/3-span repair. During the repeated loading, interfacial cracking was observed which could potentially reduce the load-bearing capacity of the concrete and thus lead to an early yield of steel.

### 3.3. NDT Results

**Figure 7:**
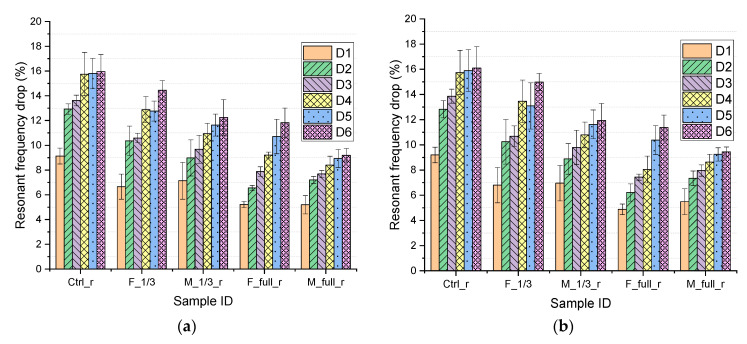
Resonant frequency for beams at different damage degree $D_{i}$ (*i* = 1,…,6) measured using (**a**) hammer percussion method (**b**) modified chain-drag method.

The NDTs give quick and easy assessments of the beam damage after each increment of repeated loading. The goal is to capture early damage that may not be visible to naked eyes. Figure 7a,b summarize the drop in resonant frequency of the repaired and control beams after repeated loading and unloading. It can be observed that after the first load step (i.e., at damage degree $D_{1}$), all beams exhibited a significant drop in resonant frequency, which accounts for approximately 5–9% of its original resonant frequency. This is due to the generation of the cracks during the loading–unloading process. Following the first load step, the development of the cracks tends to stabilize such that frequencies drops are less remarkable until the yield of the steel reinforcement [45]. Regardless of the NDT methods used, intact and repair beams experienced a drop in resonant frequency ranging from 9% to 16% at damage degree $D_{6}$. After all load steps are completed, beams having full length repairs using Mix M (M_full_r) showed the lowest drop (9%) in resonant frequency while the control mix (Ctrl_r) showed the highest drop (16%) in resonant frequency. This is consistent with flexural test results as the Ctrl_r and M_full_r showed the lowest and highest average yield load, 52,791 N and 60,147 N, respectively. Previous studies [45,46] reported that resonant frequencies reflect the changes in structural stiffness due to crack formation and thus are related to the damage severity of the structure. In this study, all specimens have the same rebar layout so the difference in yield load is mainly due to repair materials. Moreover, it is found that hammer percussion and modified chain drag method are well correlated and gave very similar NDT results.

**Figure 8:**
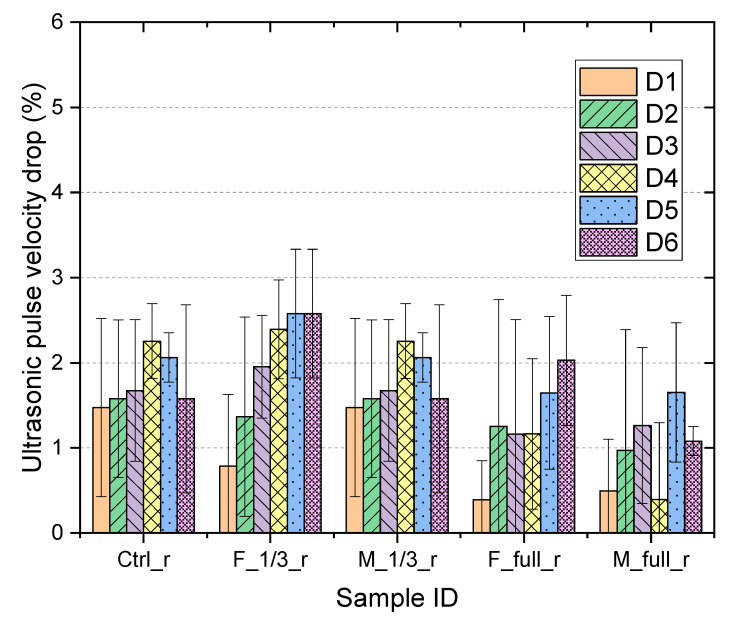
UPV of beams at different damage degree $D_{i}$ (*i* = 1,…,6).

The UPV test was performed at the center of the beam to detect debonding at the concrete-repair interface and damage caused by repeated loading. Figure 7 shows the drop in UPV for all samples at different damage degrees. An increasing drop in UPV values can be observed for all samples, which indicates the progressive damage due to repeated loading. When repeated loading was completed (at damage level $D_{6}$), samples F_1/3_r and F_Full_r showed the highest (2.6%) and second-highest (2.0%) drops in UPV respectively among all repaired samples. This is most likely due to the concrete cracking and debonding between concrete and repair. As shown in Table 4, samples repaired with cementitious repair concrete (Mix F) showed severer cracking compared to those repaired with cementitious repair mortar with modified binder (Mix M). Moreover, all beams with 1/3 span repaired with Mix F showed horizontal interfacial cracks. For sample F_1/3_r, the earliest horizontal interfacial cracks started to appear at damage level $D_{3}$ (shown in Table 4) which matches the notable drop in UPV as shown in Figure 8.

### 3.4. Load-Strain Relationship

**Figure 9:**
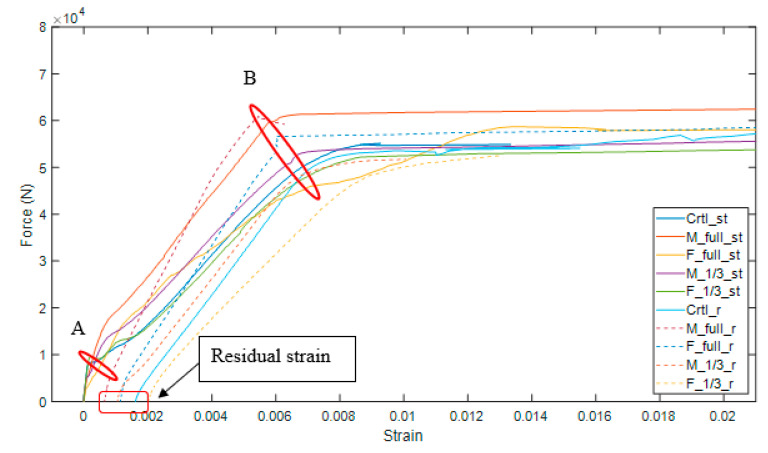
Load vs. strain of steel reinforcements.

From strain gauges mounted on the rebar, the load-strain relationships of steel reinforcements of different repaired and control beams are plotted in Figure 9. The tensile strain in the reinforced steel can be split into three distinct stages. The first stage is the elastic stage of concrete where cracking of the concrete has not yet started. At this stage, the load-strain curve is almost a vertical line because the load is majorly carried by the concrete, as shown in Figure 9 from 0 to point A. It can be observed that repaired and control samples have a first cracking load ranging from 3000 N to 7000 N. The second stage is the elastic stage of the steel reinforcement. After the cracking of concrete, the steel reinforcement starts carrying most of tensile forces. As shown in Figure 9 from point A to B, the strain in steel reinforcements begin to grow. It can be observed that the slopes of the load–strain curves decreased slightly after the cracking of concrete (point A). Stage three is the yield stage of the steel reinforcement. As shown in Figure 9, this happens when the strain in steel reinforcement significantly increases while the load only increases slightly. Compared with the beams that have gone through repeated loading, the slopes of the load–strain curves of the undamaged beams are slightly lower. This is due to the internal force redistribution for damaged beams which causes the rate of change of strain to differ between damaged and undamaged beams [47].

## 4. Theoretical Analysis of Load-Bearing Capacity

The RC beam was designed such that it is expected to fail in steel control mode. An equivalent rectangular stress block was used to replace the actual stress distribution as per CSA 23.3 [48].

The depth of the rectangular stress block was determined using Equation (1).

(1) $$a=\frac{\emptyset_{s}f_{y}A_{s}}{\alpha_{1}\emptyset_{c}f_{c}b}$$

where $\alpha_{1}$ is a mathematical parameter based on the requirement that the compression stress resultants of the actual and equivalent rectangular stress distributions are equal, $f_{y}$ is the yield strength of tensile steel reinforcement, $f_{c}$ is the compressive strength of concrete, $A_{s}$ is the reinforcement area, $\emptyset_{s}$ and $\emptyset_{c}$ are the resistance factors of steel and concrete, respectively, *b* is the width of the sample

The theoretical moment resistance ($M_{r\_th}$) of the RC beam was calculated using Equation (2).

(2) $$M_{r\_th}=\emptyset_{s}f_{y}A_{s}\left(d-\frac{a}{2}\right)$$

where *d* is the distance from the centroid of the tension steel to the extreme compression fiber.

The design guideline CSA 23.3 CL. 10.1. 7 also specifies the formula for calculating the first cracking moment of RC beams, as shown in Equations (3) and (4).

(3) $$M_{cr\_th}=\frac{f_{r}I_{g}}{y_{t}}$$

(4) $$I_{g}=\frac{bh^{3}}{12}\;$$

where $M_{cr\_th}$ is the theoretical cracking moment of the RC beam, $f_{r}$ is the modulus of rupture of the concrete on the tension side measured by flexural test (with results summarized in Table 2), $I_{g}$ is the moment of inertia of the gross cross-section of concrete beams around the neutral axis, $y_{t}$ equals h/2 which is the distance from the neutral axis to the extreme tension fiber.

To determine the moment resistance ($M_{r\_exp}$) and first cracking moment ($M_{cr\_exp}$) of a beam experimentally, Equations (5) and (6) are used, respectively.

(5) $$M_{r\_exp}=\frac{P_{y}L}{4}$$

(6) $$M_{cr\_exp}=\frac{P_{cr}L}{4}$$

Table 8 summarizes the experimental and theoretical results of moment resistance and first cracking moment of test beams. Regarding the moment resistance, the ratio of experimental values to its theoretical prediction ranges from 0.98 to 1.04 for beams without repair and with full-length repair, which indicates a good correlation. Note that for beams with a 1/3-span repair, the theoretical calculation overestimates the moment resistance by 10%. The low moment resistance could be due to the weak interfacial bond which reduces the loading bearing capacity of the repair materials. In terms of first cracking moment, the theoretical calculations have a good prediction over beams repaired with Mix F with a ratio of 0.96. However, the prediction ratio of Mix M-repaired beams ranges from 0.72 to 0.79, which means concrete cracked before the flexural strength was reached. The reasons could be manifold. Since slight interfacial cracking was observed when loading the beam with 1/3 repair, the main cause could be due to a weak interfacial bond. For beams with full-length repair, the reason could be the size effect of concrete which is caused by the low workability of the mix. The size effect happens when the strength of a concrete member decreases when its size increases. For large area repair, the repair requires good workability for sufficient consolidation.

## 5. Conclusions and Recommendations

The flexural behavior of RC beams repaired with different repair materials was investigated under both static and repeated loading conditions. The purpose of this study was to evaluate the effect of length of repair, bond strength, and compressive/flexural strength of repair on the flexural behavior of RC beams. To achieve this purpose, this study compared and analyzed the yield strength, load-displacement curve, load-strain curve before and after the repeated loading. In addition, three different NDT methods were employed to capture and estimate the material damage after each loading group of the repeated loading proposed by the authors. Based on the experimental study, the following conclusions are drawn:

1. The length of the repair affects the yield strength of repaired RC beams. The beams without vertical concrete-repair interface showed higher yield strength compared to beams having the interfaces.
2. The repair material that has higher bond strength and flexural strength is found to be beneficial to a more successful repair under static and repeated loading conditions.
3. The hammer percussion and modified chain drag methods are useful methods to detect concrete cracking and accumulated damage by measuring the resonant frequency drop after repeated loading. The resonant frequency drops are well correlated with the destructive test results such as yield strength results.
4. The formulas proposed by CSA 23.3 can effectively predict the moment resistance of both intact (control) and repaired RC beams for the geometry studied in this research. The ratio of experimental moment resistance values to its predictions ranges from 0.91 to 1.04. In addition, the formulas proposed by CSA 23.3 can predict cracking moment of control and Mix F-repaired RC beams well.
5. This research presents a preliminary study that investigated the performance of repaired RC beams under static and repeated loading conditions. One limitation of the current study is that the sample size of the repaired RC beams is small compared to beams in real structures. Future study will involve repair work and NDT measurements on real structures to validate the results. Moreover, to have a more accurate prediction on structural behavior of repaired RC beams, numerical simulations using Finite Element Method need to be developed for future studies.

## Figures and Tables

**Table 1:** Mix proportions of the parent material receiving the repair ($\mathrm{kg}/m^{3}$).

Mix ID	Cement	Sand	Gravel	Superplasticizer	AEA ^a^	Water
Mix S	450	762	1053	2.25	2.25	182.7

^a^ AEA: Air Entraining Admixture.

**Table 2:** Fresh and hardened properties of parent and repair mixes.

Mix	Hardened $\mathrm{Density}\;(kg/m^{3})$	Air Content (%)	Setting Time (Min)	*w/m* Ratio	$f_{c}^{\prime }\;\mathrm{at}\;28\;\mathrm{Days}\;(\mathrm{MPa})$	$f_{r}^{\prime }\;\mathrm{at}\;28\;\mathrm{Days}\;(\mathrm{MPa})$	Slump (mm)	Slant Shear Bond (MPa)	Splitting Tensile Bond (MPa)
Mix S	2300	3	90	0.08	45±2.7	4.8±0.4	60	*	*
Mix M	2325	5.0	75	0.09	68±2.4	7.0±0.7	70	16.7±2.3	2.3±0.6
Mix F	2374	5.1	*	0.1	55±2.2	4.9±0.6	80	5.3±7.4	1.3±0.04

* Data Not Available.

**Table 3:** Description of test specimens.

Sample ID	Repair Materials	Repair Length	Loading Protocols	Number of RC Beams
Ctrl_st	None	Intact	Static/	3
Ctrl_r	None	Intact	Repeated loading	3
F_1/3_st	Mix F	1/3 span	Static	3
F_1/3_r	Mix F	1/3 span	Repeated loading	3
F_full_st	Mix F	Full length	Static	3
F_full_r	Mix F	Full length	Repeated loading	3
M_1/3_st	Mix M	1/3 span	Static	3
M_1/3_r	Mix M	1/3 span	Repeated loading	3
M_full_st	Mix M	Full length	Static	3
M_full_r	Mix M	Full length	Repeated loading	3

**Table 4:** Summary of damage levels at which vertical and horizontal bond failure started.

Mix ID	Sample	Vertical Bond Failure Initiation Stage	Horizontal Bond Failure Initiation Stage
M_1/3_r	1	$D_{4}$	None
	2	$D_{4}$	None
	3	$D_{4}$	None
F_full_r	1	Not applicable	$D_{4}$
	2	Not applicable	$D_{3}$
	3	Not applicable	$D_{6}$
F_1/3_r	1	$D_{2}$	$D_{4}$
	2	$D_{2}$	$D_{5}$
	3	$D_{2}$	None
M_full_r	1	Not applicable	None
	2	Not applicable	None
	3	Not applicable	None

**Table 5:** Summary of crack length after repeated loading.

		Accumulative Crack Length in mm (Including Interfacial Cracks)					
Mix ID	Sample	$D_{1}$	$D_{2}$	$D_{3}$	$D_{4}$	$D_{5}$	$D_{6}$
M_1/3_r	1	80	105	116	301	407	452
	2	0	50	85	236	354	420
	3	0	32	32	132	240	367
F_full_r	1	29	86	229	360	460	485
	2	0	85	199	310	486	556
	3	0	0	136	271	520	591
F_1/3_r	1	53	140	226	292	344	470
	2	92	203	235	346	462	519
	3	54	162	218	288	423	493
M_full_r	1	29	102	201	226	252	350
	2	0	40	80	116	158	249
	3	0	0	82	262	298	397
Ctrl_r	1	187	200	290	370	423	538
	2	205	267	319	462	586	610
	3	133	211	299	372	408	503

**Table 6:** Yield strength, bending stiffness, and NDT results.

	Yield Load (N)	Resonant Frequency (Hz)		UPV through Repair Sections (m/s)
		Hammer Percussion	Modified Chain Drag	
Ctrl_st	55,504±979	671±3	670±3	4505±15
F_1/3_st	52,545±1865	587±5	589±6	4528±39
F_full_st	57,484±2007	637±3	638±3	4535±143
M_1/3_st	51,692±1210	606 ± 17	608±16	4528±39
M_full_st	59,046±1317	699±7	699±8	4579±70

**Table 7:** Yield strength of the RC beams post repeated loading.

	Yield Load (N)
Ctrl_r	52,791±722
F_1/3_r	52,909±880
F_full_r	57,269±2742
M_1/3_r	52,882±680
M_full_r	60,147±722

**Table 8:** Experimental and theoretical moment resistance and first cracking moment.

Sample ID	Moment Resistance			First Cracking Moment		
	$M_{r\_exp}$ (kN·m)	$M_{r\_th}$ (kN·m)	$M_{r\_exp}/M_{r\_th}$	$M_{cr\_exp}$ (kN·m)	$M_{cr\_th}$ (kN·m)	$M_{cr\_exp}/M_{cr\_th}$
Ctrl_st	11.1	11.4	0.98	2.4	2.7	0.89
F_1/3_st	10.6	11.4	0.93	2.7	2.8	0.96
F_full_st	11.5	11.4	1.01	2.7	2.8	0.96
M_1/3_st	10.4	11.4	0.91	2.8	3.9	0.72
M_full_st	11.9	11.4	1.04	3.1	3.9	0.79

## Data Availability

Some or all data, models, or code that support the findings of this study are available from the corresponding author upon reasonable request.
